# Current advances in the development of natural meniscus scaffolds: innovative approaches to decellularization and recellularization

**DOI:** 10.1007/s00441-017-2605-0

**Published:** 2017-03-31

**Authors:** Yunbin Chen, Jiaxin Chen, Zeng Zhang, Kangliang Lou, Qi Zhang, Shengyu Wang, Jinhu Ni, Wenyue Liu, Shunwu Fan, Xianfeng Lin

**Affiliations:** 1 Department of Orthopaedic Surgery, Sir Run Run Shaw Hospital, Medical College of Zhejiang University, Hangzhou, China; 2 Department of Orthopaedic Surgery, the First Affiliated Hospital of Wenzhou Medical College, Wenzhou, China; 3 Department of Endocrinology, the First Affiliated Hospital of Wenzhou Medical College, Wenzhou, China

**Keywords:** Meniscus, Decellularization, Biomechanical properties, Extracellular matrix, Recellularization

## Abstract

The increasing rate of injuries to the meniscus indicates the urgent need to develop effective repair strategies. Irreparably damaged menisci can be replaced and meniscus allografts represent the treatment of choice; however, they have several limitations, including availability and compatibility. Another approach is the use of artificial implants but their chondroprotective activities are still not proved clinically. In this situation, tissue engineering offers alternative natural decellularized extracellular matrix (ECM) scaffolds, which have shown biomechanical properties comparable to those of native menisci and are characterized by low immunogenicity and promising regenerative potential. In this article, we present an overview of meniscus decellularization methods and discuss their relative merits. In addition, we comparatively evaluate cell types used to repopulate decellularized scaffolds and analyze the biocompatibility of the existing experimental models. At present, acellular ECM hydrogels, as well as slices and powders, have been explored, which seems to be promising for partial meniscus regeneration. However, their inferior biomechanical properties (compressive and tensile stiffness) compared to natural menisci should be improved. Although an optimal decellularized meniscus scaffold still needs to be developed and thoroughly validated for its regenerative potential in vivo, we believe that decellularized ECM scaffolds are the future biomaterials for successful structural and functional replacement of menisci.

## Introduction

The meniscus of the knee consists in two semilunar (C-shaped) fibrocartilage structures located at the medial and lateral articular surfaces of the tibial plateau. Some of the functions performed by the meniscus are stabilization, nourishment and force distribution in the knee joint. A normal human meniscus consists of 72% water, 22% collagen and 0.8% glycosaminoglycans (GAGs), which act as lubricants in the joint (Tan and Cooper-White 2011).

Tears of the meniscus often take place during sporting events or heavy lifting and can lead to motor dysfunction. Among the 1,500,000 arthroscopic knee surgeries performed annually in the United States, around 50% are related to the meniscus (Kheir et al. 2011). Injuries of the knee meniscus are a serious medical problem, because tears in the inner avascular zone of the meniscus usually do not heal spontaneously and ultimately lead to permanent degenerative changes. This issue is addressed by partial or total meniscectomy developed by Gillquist et al. in 1982 (Gillquist et al. 1982), which is currently a principal therapeutic approach; however, it inevitably results in the destruction of the normal meniscus structure, presenting a high risk of osteoarthritis (Hoben and Athanasiou 2006). As an alternative to meniscectomy, allogeneic menisci have been used to replace the impaired structures but they carry the risk of inducing immunoreactivity together with unfavorable prognosis (Rath et al. 2001).

**Fig. 1:**
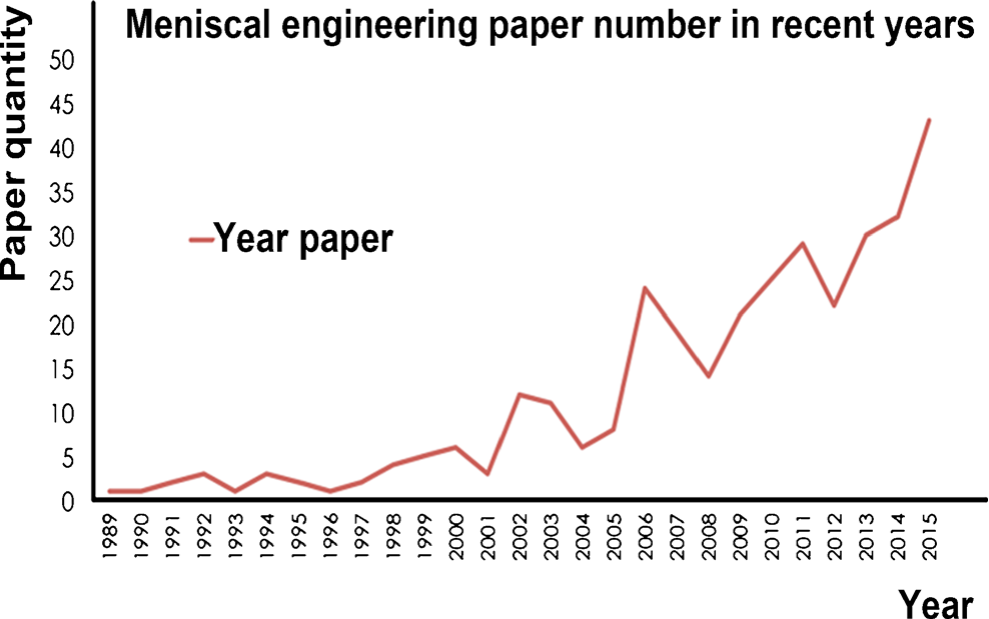
Increase in the number of publications related to meniscus engineering. Papers published from 1989 to 2015 were searched in PubMed using a key word “meniscal engineering”

Synthetic and biological materials such as the collagen meniscus implants (Stone et al. 1992, 1997), polyurethane meniscus implants (Verdonk et al. 2011), silk fibrous protein scaffolds (Mandal et al. 2011a), polycaprolactone scaffolds (Baker et al. 2010, 2012) and polyester–carbon cell-free implants (Wood et al. 1990) have been used to simulate the architecture and function of the meniscus (Scotti et al. 2013). Research activity in this direction is reflected in recent publications (Fig. 1). The difference between these materials and natural menisci lies in completely different organization and interactions among the major tissue constituents: water, GAGs and collagen. For example, coarse and circumferential collagen type I, which constitutes 98% of meniscal collagen, has a characteristic organization and orientation in the natural menisci and gives the tissue great tensile stiffness and strength (Fithian et al. 1990). Since the natural meniscus has such a complex three-dimensional structure and unique biomechanical properties as well as biological characteristics, the optimal biomaterials for the meniscus replacement still need to be developed.

**Fig. 2:**
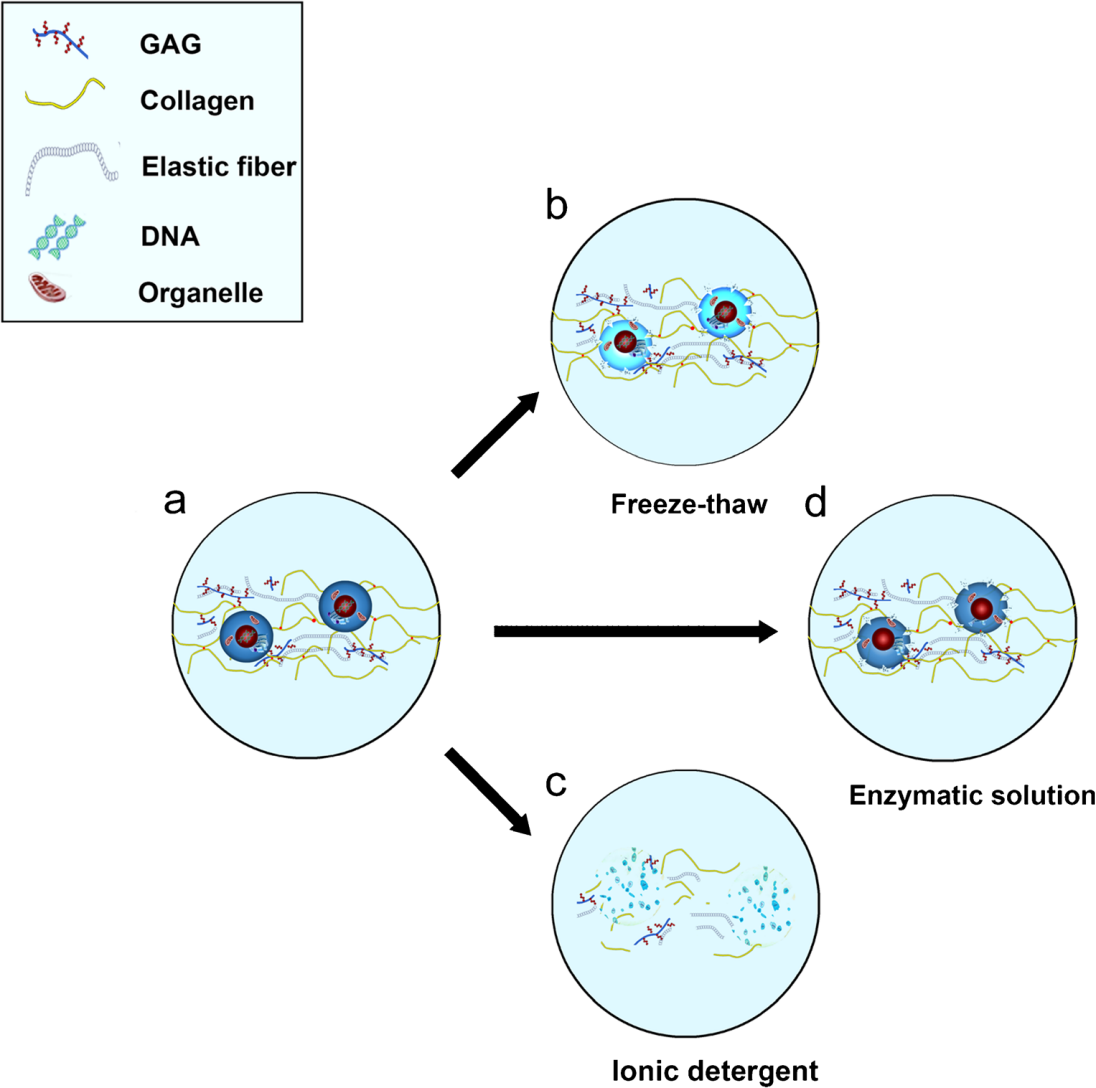
*A* Normal meniscus; normal human meniscus is composed of 72% water, 22% collagen and 0.8% glycosaminoglycans (GAGs) (Tan and Cooper-White 2011).* B* Meniscus after physical treatment (e.g., freeze–thaw cycles); formed intracellular ice crystals disrupt cellular membranes, causing cell lysis.* C* Meniscus after chemical treatment (e.g., ionic detergent); significant removal of nuclear debris and cytoplasmic proteins; however, adverse effects such as destruction of GAGs and collagen are also prominent.* D* Meniscus after enzymatic treatment (e.g., DNase); considerable DNA degradation and removal of ECM components

**Fig. 3:**
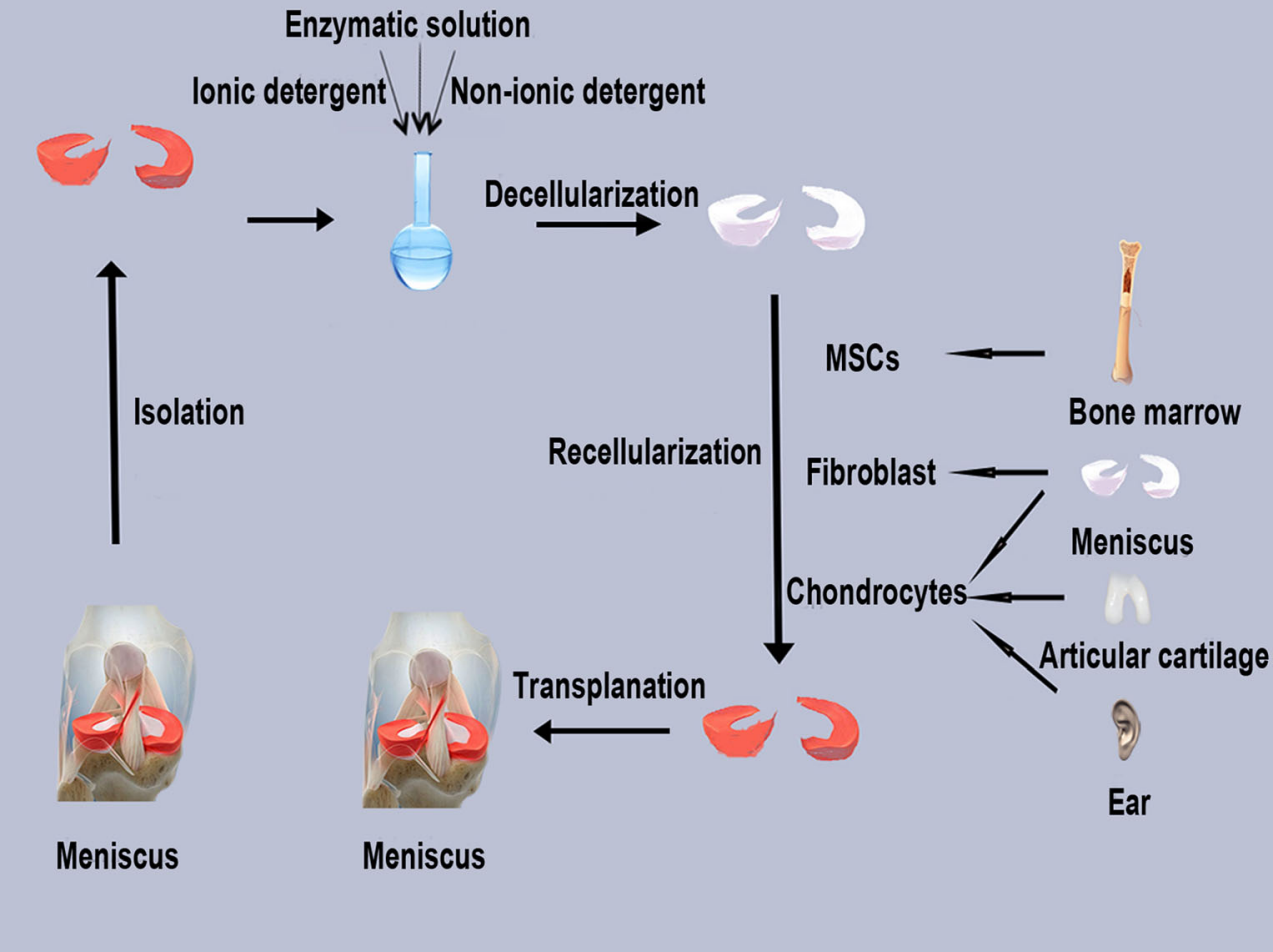
Fabrication of a cell-seeded meniscal scaffold

Nowadays, decellularized ECM scaffolds are increasingly investigated as a natural replacement of the injured meniscus, which would facilitate its regeneration. Decellularized ECM is prepared by removing cells and their components from allogeneic or xenogeneic donor tissues to produce a minimally immunogenic scaffold with required biomechanical and biological properties (Crapo et al. 2011). Cells are lysed using physical (e.g., shocks and freeze–thaw cycles), chemical (e.g., detergents such as Triton X-100 and SDS) and enzymatic (e.g., DNase and trypsin) treatments that disrupt and solubilize both the cytoplasmic and nuclear membranes of the cell (Fig. 2). The obtained decellularized scaffolds are then used to replace damaged menisci in the knee joint where they will interact with the biological environment by promoting cell infiltration and production of the ECM. As a result, they should integrate with the surrounding tissue. Thus, the general procedure of meniscus regeneration using natural ECM scaffolds includes three steps: decellularization of donor tissue ECM, repopulation with appropriate cells and implantation, i.e., remodeling and repair of the degenerated meniscus (Fig. 3).

Currently, a variety of decellularization and recellularization protocols used in meniscus replacement have been reported but there is no consensus on the optimal procedure to generate decellularized menisci or their application. This review summarizes recent advances in the research on meniscal decellularization and recellularization during the production of ECM scaffolds, which may help to create an optimal protocol and establish the strategy to be used for successful replacement and repair of injured menisci.

## Comparison of different therapeutic measures

In view of the limited healing capacity of meniscal injuries, the preservation, repair, reconstitution and replacement of meniscal tissues are indispensable. For many years, partial and total meniscectomy remained the most commonly performed orthopedic surgeries (Stabile et al. 2010) but they have several drawbacks. Natural menisci could dissipate part of the load to the chondral surface and reduce biomechanical wear; in addition, the knee stability depends on the integrity of menisci. Furthermore, meniscal injuries may induce inflammatory responses and degenerative processes (Kaleka et al. 2014).

Therefore, efforts are focused on meniscal preservation; however, not all meniscal tears are reparable and meniscus allograft transplantation is widely applied. The types of allografts used include fresh, deep-frozen, freeze-dried and cryopreserved (frozen in cell-preserving solution) (Stabile et al. 2010). Among them, long-term freezing and freeze-drying could destroy viable cells and denature histocompatibility antigens (Arnoczky et al. 1988). An allograft meniscus typically shows invasion of blood vessels and complete healing in the periphery, together with the presence of fibrochondrocytes throughout the meniscus; however, more than 50% (*P* < 0.05) reduction in the number of fibrochondrocytes has been commonly observed (Rath et al. 2001). Even after freezing, meniscal allograft transplants are potentially immunogenic, resulting in even higher post-transplantation failure rate compared to fresh grafts (Siegel and Roberts 1993; Verdonk et al. 2005). In addition, the high cost of tissue grafts, necessity of surgical precision and the risk of bacterial contamination impose restrictions on the clinical application (Kaleka et al. 2014).

Among the newly developed tissue engineering scaffolds, the collagen meniscus implant (CMI), first developed for clinical use in the United States (Stone et al. 1990), seems to be the best. Its advantages include low immunogenicity, induction of tissue regeneration, adaptable pore size and remodeling capacity of ingrown tissue (Scotti et al. 2013); nevertheless, it has a reoperation rate of 22% and a considerable degradation rate of the scaffold (from 6 months to 2 years) (Scotti et al. 2013). In addition, its inferior biomechanical properties (compressive and tensile stiffness) compared to native meniscus make the load distribution in the knee minimal or absent (Buma et al. 2007).

Under such circumstances, many researchers have focused on the development and application of decellularization technologies. A number of acellular scaffolds and related decellularization protocols have received regulatory approval for clinical use, such as human dermis (Chen et al. 2004), blood vessels (Dahl et al. 2003; Uchimura et al. 2003) and porcine heart valves (Bader et al. 1998). Decellularization has shown its particular advantages in regard to minimization of immunogenicity and preservation of the ECM, which is essential for the regeneration of organic injuries (Song and Ott 2011). The ECM secreted by resident cells of each tissue has been confirmed to provide cues affecting cell migration, proliferation, differentiation and host tissue remodeling (Crapo et al. 2011; Valentin et al. 2010; Xu et al. 2010). A good meniscus ECM scaffolds should maintain the three-dimensional structure and composition of the ECM. Unlike the CMI, the ECM scaffold which act as a frame for tissue regeneration by endogenous cells, would be effective in retaining the mechanical properties (compressive stress and tensile stiffness) of natural menisci (Buma et al. 2007; Sandmann et al. 2009; Stabile et al. 2010); as a result, biological functions could be preserved after transplantation. Indeed, after decellularization, residual cellular components could negate tissue remodeling of biologic scaffolds in vivo (Brown et al. 2009; Zhang et al. 2010). Therefore, tissue processing methods, including decellularization and recellularization, are pivotal for clinical success.

## Current protocols of meniscus decellularization

In 2005, Yamasaki et al. (2005) conducted the first in vitro study with the aim to resolve the problems of biomechanical failure, cellular toxicity and immunological reactivity reported for allogeneic transplants. In their study, awrat menisci were treated with Na-EDTA and ethanol for 3 days and freeze–thawed three times using liquid nitrogen to destroy meniscus chondrocytes; the prepared scaffolds had the potential for cellular repopulation with adequate stiffness. However, the procedure did not result in complete decellularization because allogeneic cells and their components could not be washed out and, consequently, could elicit severe immune response.

In 2007, Maier et al. (2007) investigated decellularized ovine menisci. Ovine meniscus tissues were incubated at 37 °C with agitation (120 rpm) in enzymatic solution containing 0.25% trypsin, 3 mg collagenase A (specific activity > 0.15 U/mg), 0.375 mg/ml protease and 0.02% EDTA. Histological and immunohistochemical analyses indicated complete cell removal and the absence of histocompatibility complex (MHC) I or II expression. In addition, the scaffolds had a loose structure with gaps and micropores (approximately 5–25 mm), indicating the ability of collagenase to create micropores in tissues at low concentrations. However, GAGs, which play a pivotal role in the regulation of water content within the meniscus, were partially removed (*P* < 0.01) and the disruption and partial digestion of the ECM was significant.

In 2008, Stapleton et al. (2008) published a study on the decellularization of porcine menisci that were first subjected to three dry freeze–thaw cycles for cell lysis and then incubated in hypotonic (10 mM Tris-HCl) buffer with 0.1% ionic detergent (SDS) for 48 h to solubilize cellular fragments and finally with DNase and RNase to digest nucleic acids. Nevertheless, H&E staining indicated cell presence, although cell density decreased with the distance from the vascularized region of the meniscus and there was a 59.4% loss of GAG content (*P* < 0.05). However, no significant decrease in the content of collagen (*P* > 0.05), which is responsible for the well-preserved tensile properties of the meniscal tissue, was observed (Aspden et al. 1985; Courtman et al. 1994). The decellularized scaffolds retained the tensile properties of the natural meniscus, were not cytotoxic and demonstrated good biocompatibility (Stapleton et al. 2011).

In 2009, Sandmann et al. (2009) published a study on the generation and characterization of acellular human meniscus scaffolds. In their protocol, menisci were incubated for different time periods (1 and 2 weeks) in solutions containing various concentrations of SDS (1, 2 and 5%). Complete cell removal was achieved in 2% SDS after 2 weeks without compromising compressive properties. Indeed, SDS and other detergents such as Triton X-100 could remove cells completely but SDS was more efficient in cell lysis than Triton X-100; however, the former was observed to cause extreme fragmentation and swelling of collagen fibers and was less supportive of cell reseeding of the scaffolds (Bodnar et al. 1986; Courtman et al. 1994). Thus, it is necessary to evaluate the in vivo performance of the scaffold.

In 2013, Azhim et al. (2013) developed a novel decellularization approach based on sonication. Bovine menisci were cut into fragments (10 × 10 × 5 mm) and sonicated in constantly circulating 0.1% SDS solution for 10 h in a reactor. Since sonication facilitated the penetration of SDS solution inside the samples, almost all cell nuclei could be removed by the treatment, which was superior to the immersion method used in the control group. The disadvantage was that sonication caused GAG denaturation resulting in a decrease in water content and mechanical strength.

In 2010, Stabile et al. (2010) investigated a procedure to obtain acellular allograft-derived scaffolds from ovine menisci, which was previously applied to tendon tissue (Whitlock et al. 2007). Ovine menisci were incubated in solution containing 0.05% trypsin-EDTA, 2% Triton X-100 and 1.5% peracetic acid for 48 h to remove cellular debris and nuclear components and increase tissue porosity. Histological analysis revealed the absence of nuclei in the scaffold and a 55% decrease in DNA content compared to the native menisci (*P* < 0.003). The authors suggested that residual DNA would not promote immune response but other cellular components might (Khoury et al. 1994; Rodeo et al. 2000). To analyze biocompatibility, the scaffolds and latex specimens, a positive control for cytotoxicity testing, were cultured on subconfluent monolayers of murine embryonic fibroblasts (NIH 3T3 cells) and analyzed for the viability and proliferation of infiltrating cells. More cells populated the scaffolds compared to latex structures, indicating lower cellular toxicity. Scanning electron microscopy (SEM) showed that pore connectivity of the ECM scaffolds increased from 41 to 87% (*P* < 0.01), revealing overall elongation of pathways for cell migration into the graft. However, the ideal pore size and connectivity for the biological integration of scaffolds into meniscal tissue remains unknown.

In 2011, Yu et al. (2011) reported another protocol to prepare decellularized scaffolds from rabbit menisci. Tissues were incubated in hydrogen peroxide for 1 h, 6% Triton X-100 for 24 h, 6% sodium deoxycholate for 24 h and 3% Triton X-100 for 24 h. Histological analysis indicated complete cell removal and retention of intact collagen type I.

In 2015, Chen et al. (2015) developed an acid-based decellularization method for porcine menisci, which were treated with acetic, formic, 15% peracetic, 60% malic, succinic, or 60% citric acids and freeze-dried to fabricate the scaffold. The treatment with formic acid decreased DNA content to 4.10 ± 0.03% after 2 h (*P* < 0.001) and to 0.40 ± 0.02% after 12 h, while that with acetic, peracetic, malic and citric acids caused a less significant reduction after 2 h, indicating effective decellularization with formic acid. Although formic acid reduced collagen content to 37.09 ± 0.29% (*P* = 0.011) after 12 h, it produced no adverse effect on either GAGs or collagen after 2 h while providing complete cell removal and, thus, was superior to other acids. The porosity of the acellular ECM scaffold was 85.76 ± 2.80% and no cytotoxicity was observed.

Wu et al. (2015) attempted to convert solid meniscus ECM scaffolds into injectable hydrogel. Porcine menisci were cut into 1-mm slices, frozen at 80 °C, powdered, stirred in 1% SDS in PBS for 72 h and treated with 0.1% EDTA in PBS for 24 h. To prepare the injectable hydrogel, the decellularized meniscus matrix ground to fine powder was suspended in pepsin/0.01 M HCl solution at 15 mg/mL, injected into a cylinder mold and placed in a 37 °C incubator for 30 min to form solidified hydrogel. The remolded decellularized meniscus presented a pink matrix without visible nuclear dots by H&E and DAPI staining and had a collagen content of 78 ± 22% (dry weight), which was higher than that in the native meniscal tissue (42 ± 10%). Nevertheless, the GAG content significantly decreased from 14.95 ± 5.57 μg/mg to 0.54 ± 0.08 μg/mg.

## Maintenance of biomechanical properties

A major consideration in the preparation and evaluation of the ECM scaffolds for meniscal repair and replacement is the preservation and restoration of the mechanical function. Biomechanical factors play a pivotal role in the design and synthesis of tissue-engineered biomaterials and are also important for evaluating the efficacy of restoring normal meniscal function (Setton et al. 1999). The microstructure of the meniscus is optimal for load support in the knee and a high density of collagen fiber bundles (primarily type I collagen) allow the sustaining of the tensile stress generated during functional loading (Aspden et al. 1985; Bullough et al. 1970).

Among biomechanical properties, the most important is viscoelasticity characterized by three important parameters: compressibility, relaxation and stiffness (Maier et al. 2007), measured by cyclic compression–relaxation tests. Briefly, the sample is preloaded and compressed dynamically with constant velocity, then compressed statically and relaxed with constant velocity. The “compressibility” is defined as the end of the dynamic compression phase, while the “residual force” is defined as the end of the static compression phase and the “stiffness” is calculated from the linear-elastic slope of loading (Maier et al. 2007).

After the enzymatic digestion process conducted by Maier et al. (2007), GAG extraction changed the structure of the ECM, increasing compressive stiffness and compressibility and slightly decreasing the residual force. The results were quite similar to those obtained by Azhim et al. (2013), who also detected GAG denaturation after sonication. The extraction of GAGs resulted in the loss of water (Schmidt et al. 1990; Zhu et al. 1993) and thus contributing to the increase of stiffness, which was described by Maier et al. (2007). Abdelgaied et al. (2015) followed the same protocol developed by Stapleton et al. (2008) to compare tensile and compressive properties of decellularized and normal porcine menisci. The decellularized menisci showed lower compressive elastic modulus and higher compressive permeability compared to the natural structure, which could be attributed to a 60% loss of GAG content.

On the other hand, Sandmann et al. (2009) characterized their scaffold and verified preservation of the compressive properties. Thus, it can be suggested that SDS would not destroy GAGs to the same extent as sonication and enzymatic digestion. Stabile et al. (2010) also confirmed the successful maintenance of the compressive and tensile biomechanical properties in the meniscus scaffold they created. It can be concluded that the chemical reagents such as Triton X-100 and 1.5% peracetic acid used in their protocol may present milder and more effective treatment to retain the microstructure of menisci.

In the future, in-depth investigation of full meniscus ECM scaffolds in animal models is essential, since the ultimate goal is to test biological and physical functions of the scaffolds in vivo.

## Potential source of cells for the recellularization of meniscus ECM scaffolds

Meniscal tissue contains fibroblast-like cells located at the outer vascular region and round fibrochondrocytes interspersed within the middle and inner regions (Hasan et al. 2014; Rongen et al. 2014).

In tissue engineering, several types of cells have been used for recellularization of artificial and natural biological scaffolds. Cell-seeded constructs offer potential advantages by enhancing the integration of scaffolds with native tissues and providing a specific cellular microenvironment to promote required cellular activities, including ECM production, cell proliferation and activation of cell signaling pathways. In decellularized menisci, ideal cells for recellularization should be minimally immunogenic, easily obtainable and able to generate the native ECM.

Although meniscal fibrochondrocytes are the most common cells in the meniscus and are easy to extract, their proliferative capacity in humans decreases dramatically with age (Barbero et al. 2004). In an early study on scaffold recellularization, fibrochondrocytes were seeded on polyglycolic acid structures and implanted into nude mice (Ibarra et al. 1997). At first, only granulation tissue was formed, while histologically fibrocartilaginous tissue was developed later. However, the production of the ECM remained a challenge.

In the first study on recellularization of decellularized meniscus scaffolds, Maier et al. (2007) seeded autologous fibrochondrocytes onto ovine meniscus scaffolds using a manual needle injection technique. Cells survived and proliferated for over 28 days, demonstrating the feasibility of culturing cells within ECM scaffolds. It was the first study to seed meniscal cells into an allogeneic meniscus scaffold that survived for such a long time. However, cell differentiation and ECM production were not investigated.

In another study, decellularized ECM scaffolds were seeded with human primary chondrocytes, which were dropped directly into the scaffolds using a pipette (Chen et al. 2015). To measure DNA, GAGs and total collagen contents, samples were digested with papain. Chondrocytes were cultured on the scaffold for 7, 14, 21 and 28 days and DNA content began to show 1.03-, 1.89- and 2.62-fold increases, respectively, after 14, 21 and 28 days. In addition, cell numbers increased 10.1-fold over 28 days. Total collagen content was assessed by acid hydrolysis and treatment with chloramine-T and p-dimethylaminobenzaldehyde in papain-digested samples to determine hydroxyproline, which was converted to total collagen using a mass ratio of 7.25 (Pal et al. 1981). GAGs, total collagen and type II collagen synthesis by scaffold-seeded cells increased by 572.34%, 301.11% and 191.79% from day 7 to day 21, respectively. However, the content of type I collagen was below the detection limit [lower limit of quantification (LLOQ) < 0.08 μg/mL]. These data indicated the deposition and de novo synthesis of GAGs, total collagen and type II collagen by chondrocytes cultured on acellular ECM scaffolds. The results were in agreement with another study showing that auricular chondrocytes may have a greater capacity for the synthesis of type II collagen and GAGs than fibrochondrocytes (Hoben et al. 2007). Since the meniscus contains approximately 98% type I collagen (Sun et al. 2012), it can be suggested that auricular cartilage may not be an optimal cell source for meniscus acellular scaffolds.

In addition, mesenchymal stem cells (MSCs), which have been proved to function as pluripotent cell progenitors, may be a good choice for cartilage regeneration (Arnoczky 1999; Wakitani et al. 1994). Yamasaki et al. (2008) reported that bone marrow-derived MSCs seeded on the scaffolds showed a chondroprotective effect in a rat model. When human (h)MSCs were cultured with 3D aqueous silk-derived scaffolds fabricated by salt-leaching and freeze-drying to mimic native meniscus pore heterogeneity (Mandal et al. 2011a), they demonstrated a significant upregulation of cartilage-related ECM markers such as collagen type I, aggrecan and SOX-9 (Mandal et al. 2011b). Another study indicated that mature meniscal cells cultured with hMSCs at a 3:1 ratio showed higher expression of collagen type I and GAGs and lower levels of hypertrophic biomarkers such as collagen X and MMP13, demonstrating a more pronounced meniscal phenotype compared to pure hMSC cultures (Cui et al. 2012).

Dermal fibroblasts could also be chosen for differentiation to fibrochondrocytes. Indeed, the transfection with the genes encoding SOX proteins 5, 6 and 9, co-culture with demineralized bone powder and growth on aggrecan- or perlecan-coated surfaces have been proved to upregulate collagen II and GAG synthesis in fibroblasts (French et al. 2002, 2004; Ikeda et al. 2004, Mizuno and Glowacki 1996).

Stapleton et al. (2011) incubated porcine medial meniscal cells (PMMCs) and human dermal fibroblasts (PHDFs) on decellularized meniscus scaffolds. The approximate confluent cell density (CCD) was 4 × 10^3^ cells/cm^2^ for PHDFs and 6 × 10^3^ cells/cm^2^ for PMMCs. PHDFs seeded at the CCD showed limited attachment and some of the adhered cells were not fully spread on the tissue surface as evidenced by SEM analysis. At the seeding density of 10 × CCD, cellular attachment was improved and at 100 × CCD, cells formed a complete monolayer across the tissue surface. However, no cell infiltration into the inner compartments of the acellular scaffold was detected after 24 h. PMMCs seeded at the CCD showed attachment to the scaffolds and the majority of cells exhibited flattened morphology; however, infiltration was also not observed.

These decellularized meniscus scaffolds failed to be recellularized as full-thickness menisci partially because of their dense structure. Wu et al. (2015) seeded primary bovine chondrocytes isolated from knee joints of calves on the surface of meniscus-derived ECM hydrogel together with fibroblasts. At day 14, 21 ± 4% of chondrocytes were observed in a 1200–1500-μm zone as shown by DAPI labeling, where they demonstrated even distribution, round morphology and positive SOX9 staining. In addition, the hydrogel showed good tissue compatibility in vivo.

## In vivo immunobiocompatibility

The rejection of animal tissues transplanted to humans mostly occurs due to hyperimmunoreactivity of the host towards the scaffold or its degradation products, which may be mediated by MHC class I and II. Maier et al. (2007) conducted immunohistochemistry analysis of MHC before and after meniscus processing. Specific positive reactivity was observed for synovial and endothelial cells (MHC II) and fibrochondrocytes (MHC I) in the native meniscus, while none was observed after decellularization.

Furthermore, antibodies against galactose-α-1,3-galactose (α-Gal) and continuous antigenic cross-reaction with gastrointestinal bacteria (Galili 2001) may also cause hyperimmune rejection. Therefore, it is important to ensure the removal of α-Gal epitopes from the meniscus intended for clinical application. To determine the residual α-Gal content after decellularization, Stapleton et al. (2008) used α-galactosyl transferase-deficient GTKO mice, demonstrating more significant immunoreactivity against the α-Gal epitopes abundant in menisci. Mice were immunized or not with porcine blood cells and were implanted subcutaneously with fresh, decellularized, or decellularized α-galactosidase-treated meniscal tissues. As a result, mice demonstrated 240, 480 and 1260 antibody units to fresh, decellularized and α-galactosidase-treated menisci, respectively, suggesting the removal of α-Gal epitopes through decellularization. Immunohistochemistry revealed no signs of immunoreactivity in decellularized scaffolds. The scaffolds tended to be acellular in the center, while the periphery of the explants was populated mostly with fibroblast-like cells. The explants were surrounded by fibrous capsules containing mononuclear cells, which were thicker than the untreated native menisci. The data suggested that in vitro recellularization before implantation may result in a loss of the donor cells after implantation, which was consistent with the results of another study (Jackson et al. 1993). Therefore, the in vivo recellularization approaches may need to be developed (Ionescu and Mauck 2013).

Chen et al. (2015) evaluated the immunocompatibility of decellularized ECM scaffolds in rats. The scaffolds were implanted dorsally into four subcutaneous pockets and analyzed for inflammatory reactions on days 7, 14 and 28 post-implantation. In contrast to native implants, no signs of inflammation were observed for decellularized scaffolds that were absorbed at day 28, indicating that the decellularization of allogeneic menisci could significantly reduce immune reactivity in vivo.

In another study, surgical trauma was minimized by a subcutaneous injection of meniscus-derived ECM hydrogel that was solidified into stable opaque hydrogel 30 min after implantation (Wu et al. 2015). Over the next week, the formed hydrogel decreased in volume and was infiltrated by granulocytes and macrophages; at day 3, cell percentage in the 200- to 300-μm zone increased from 4 ± 1% to 27 ± 9%. No apparent angiogenesis was observed in the tissue surrounding the scaffold.

## Discussion

**Fig. 4:**
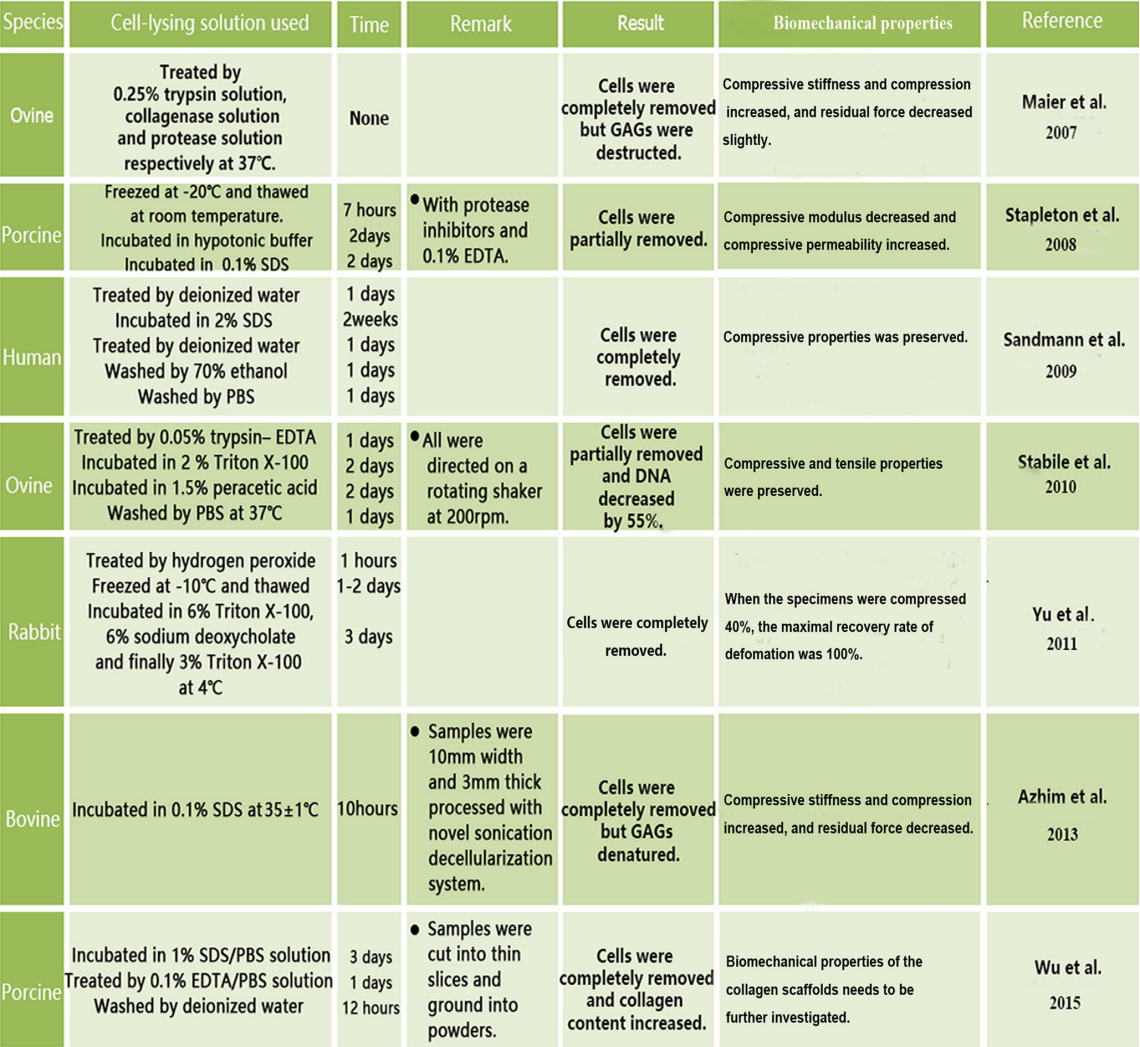
Current decellularization strategies for the meniscus

Menisci from rats, sheep, pigs and humans could be decellularized using a variety of methods, including physical (freezing–thawing, sonication), chemical (detergents such as SDS, peracetic acid and Triton X-100; EDTA, hypotonic buffers), biological (enzymatic digestion) and their combinations (Fig. 4).

Methods such as freezing–thawing and incubation with detergents or hypotonic buffers (Tris-HCl) can be used for cell lysis without significant adverse effects on the meniscal ECM (Stapleton et al. 2008). Sonication and detergents can also destroy the nuclei, which may be an advantage (Gilbert et al. 2006). The treatment with collagenase can create micropores within menisci but at the same time may denature GAGs (Maier et al. 2007). Although SDS has been proved to effectively remove cells while retaining collagen fibers, it separates GAGs from proteins in the ECM and residual SDS presence may have adverse effects on cell growth and tissue regeneration (Wu et al. 2015). Therefore, the time of SDS treatment should be reduced and milder detergents should be considered for meniscus decellularization to ensure GAG retention. Similarly, sonication may also cause GAG denaturation (Azhim et al. 2013), while formic acid, which was proved to be effective in decreasing DNA content, may destruct collagen (Chen et al. 2015). According to previous studies on meniscal decellularization, tensile properties are mostly attributed to collagen fibers, while compressive properties are mostly associated with GAGs (Abdelgaied et al. 2015; Maier et al. 2007; Schmidt et al. 1990; Zhu et al. 1993). In addition, GAGs colocalized with type II collagen could maintain viscoelastic properties, compression stiffness and tissue hydration (Buma et al. 2007), while a 1% decrease in GAG content could result in a 1.1% decrease in elasticity (Zwierzchowski et al. 2015). Since meniscal biomechanical properties are closely related to the structure and organization of the collagen network and GAG content, the ECM retaining these components after decellularization would, at the same time, possess favorable biomechanical characteristics.

Among all investigated protocols, 2% SDS treatment of whole human menisci for 2 weeks preserved compressive properties (Sandmann et al. 2009), while 2% Triton X-100 and 1.5% peracetic acid treatment of whole ovine menisci for 48 h preserved both compressive and tensile characteristics (Stabile et al. 2010) and collagenase and protease treatment of whole ovine menisci resulted in the increase of compressive stiffness and compressibility (Maier et al. 2007). As for partial meniscal decellularization, bovine menisci processed by sonication also increased compressive stiffness and compressibility (Azhim et al. 2013), while porcine menisci incubated in 0.1% SDS for 2 days showed a decrease in compressive modulus and an increase in compressive permeability (Abdelgaied et al. 2015) (Fig. 4). Thus, it can be suggested that enzymatic digestion and sonication as well as SDS processing may influence the biomechanical features of decellularized menisci.

**Fig. 5:**
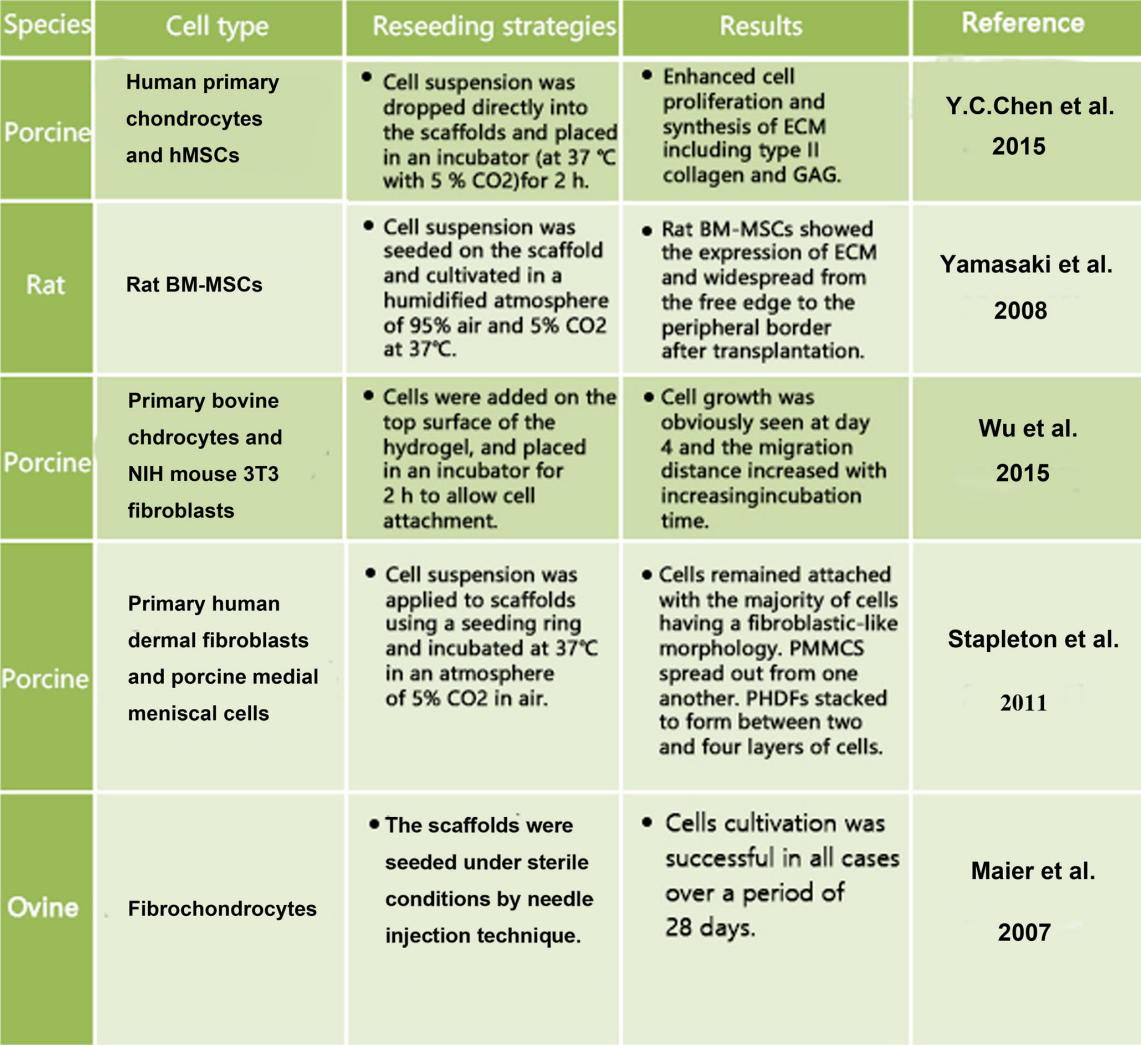
Cell sources for recellularization

Regarding the recellularization of ECM scaffolds, several cell types have been investigated, including fibrochondrocytes, primary chondrocytes, MSCs and fibroblasts (Fig. 5). Among them, fibrochondrocytes have been proved to dramatically decrease their proliferative capacity with donor age. Chondrocytes and fibroblasts have been applied to assess cellular compatibility of the scaffolds (Peretti et al. 2001, 2004). In addition, bone-marrow (BM)-derived MSCs have been suggested as an excellent cell type for seeding into acellular meniscus scaffolds (Yamasaki et al. 2008). BM-MSCs showed a good infiltration rate and expressed specific ECM components. In addition, hMSCs have also been a good choice to study the ability of scaffolds to promote chondrogenic differentiation and increase the expression of genes encoding aggrecan and type II collagen. Co-culture of meniscal cells with hMSCs at a 3:1 ratio promoted the highest synthesis of molecules essential for the maintenance of meniscus integrity (Cui et al. 2012). However, long-term evaluation of the transplanted tissues is required to validate the clinical potential of BM-MSC-seeded scaffolds.

Most studies have indicated that the ECM scaffolds have good immunocompatibility with recipient tissues due to complete removal of donor cells (Chen et al. 2015; Stapleton et al. 2011). However, the ECM of meniscal tissues has a thick and dense fibrous structure (Stapleton et al. 2008), which may be a problem for cell infiltration into the inner region of the implanted scaffolds. The solution for this problem may be processing acellular scaffolds into hydrogels, which results in increased porosity within the implanted material and rapid cell infiltration (Wu et al. 2015). Thus, decellularized meniscal tissues with low immunogenicity and improved porosity, such as meniscal slices, powders and hydrogels could be more effective in regard to recellularization compared with intact acellular meniscal scaffolds. However, the processed decellularized ECM scaffolds may have a disadvantage of losing biomechanical properties of the native structure, such as stress and tension resistance, which could in turn affect cellular behavior and metabolic activity in vivo; therefore, their application may be limited to partial meniscus regeneration. Further studies should be conducted to investigate the regenerative capacity of processed ECM scaffolds in vivo and their applicability to the repair of partial defects in menisci.

In conclusion, the decellularized meniscus scaffolds have demonstrated good biocompatibility, biomechanical characteristics and regenerative properties and present a promising approach to the functional restoration of injured menisci.
